# Evaluating Magnetic Resonance Spectroscopy as a Tool for Monitoring Therapeutic Response of Whole Brain Radiotherapy in a Mouse Model for Breast-to-Brain Metastasis

**DOI:** 10.3389/fonc.2019.01324

**Published:** 2019-11-27

**Authors:** Woon Hyung Chae, Katja Niesel, Michael Schulz, Florian Klemm, Johanna A. Joyce, Marcus Prümmer, Boris Brill, Judith Bergs, Franz Rödel, Ulrich Pilatus, Lisa Sevenich

**Affiliations:** ^1^Georg-Speyer-Haus, Institute for Tumor Biology and Experimental Therapy, Frankfurt, Germany; ^2^Faculty of Biological Sciences, Goethe-University, Frankfurt, Germany; ^3^Department of Oncology and Ludwig Institute for Cancer Research, University of Lausanne, Lausanne, Switzerland; ^4^Chimaera GmbH, Erlangen, Germany; ^5^Department of Radiotherapy and Oncology, University Hospital Frankfurt, Goethe University, Frankfurt am Main, Germany; ^6^German Cancer Consortium (DKTK) and German Cancer Research Center (DKFZ), Heidelberg, Germany; ^7^Frankfurt Cancer Institute, Goethe University, Frankfurt am Main, Germany; ^8^Institute of Neuroradiology, University Hospital Frankfurt, Goethe University, Frankfurt am Main, Germany

**Keywords:** radiotherapy, brain metastases, magnetic resonance imaging, spectroscopy, metabolites, radio-response, pre-clinical models

## Abstract

Brain metastases are the most common intracranial tumor in adults and are associated with poor patient prognosis and median survival of only a few months. Treatment options for brain metastasis patients remain limited and largely depend on surgical resection, radio- and/or chemotherapy. The development and pre-clinical testing of novel therapeutic strategies require reliable experimental models and diagnostic tools that closely mimic technologies that are used in the clinic and reflect histopathological and biochemical changes that distinguish tumor progression from therapeutic response. In this study, we sought to test the applicability of magnetic resonance (MR) spectroscopy in combination with MR imaging to closely monitor therapeutic efficacy in a breast-to-brain metastasis model. Given the importance of radiotherapy as the standard of care for the majority of brain metastases patients, we chose to monitor the post-irradiation response by magnetic resonance spectroscopy (MRS) in combination with MR imaging (MRI) using a 7 Tesla small animal scanner. Radiation was applied as whole brain radiotherapy (WBRT) using the image-guided Small Animal Radiation Research Platform (SARRP). Here we describe alterations in different metabolites, including creatine and N-acetylaspartate, that are characteristic for brain metastases progression and lactate, which indicates hypoxia, while choline levels remained stable. Radiotherapy resulted in normalization of metabolite levels indicating tumor stasis or regression in response to treatment. Our data indicate that the use of MR spectroscopy in addition to MRI represents a valuable tool to closely monitor not only volumetrical but also metabolic changes during tumor progression and to evaluate therapeutic efficacy of intervention strategies. Adapting the analytical technology in brain metastasis models to those used in clinical settings will increase the translational significance of experimental evaluation and thus contribute to the advancement of pre-clinical assessment of novel therapeutic strategies to improve treatment options for brain metastases patients.

## Introduction

Brain metastases are the most common brain tumor in adults with an incidence of 10 per 100,000 population across all tumor types (1). Melanoma, breast- and lung cancer are the type of cancers that most frequently metastasize to the brain (2, 3). Brain metastasis is associated with poor prognosis and deteriorated quality of life due to headaches, epileptic seizures, and gradual cognitive impairment in addition to possibly advanced primary disease and dissemination to other organs (4). Improved management of primary cancers associated with prolonged patient survival has even led to an increase in the incidence of brain metastases with minimal progress in available therapeutic modalities for cerebral lesions (5, 6). Despite the known side effects of radiotherapy such as acute or delayed neurotoxicity and loss of memory function as well as radiation necrosis (7, 8), whole brain radiotherapy (WBRT) or stereotactic radiosurgery (SRS) still represent one of the mainstays of therapeutic intervention for brain metastases patients (7). However, detecting potential structural side effects of radiotherapy such as radiation necrosis is challenging due to its similarity to recurrent brain tumor in conventional imaging methods (9). Consequently, several studies focused on MR spectroscopy as an alternative to MRI and computer tomography (CT). MR spectroscopy provides a metabolic spectrum of a select area instead of presenting the morphology of these structures (10). Characteristic metabolic changes such as an increase in the ratio of choline (Cho) to creatine (Cr) and Cho to N-acetylaspartate (NAA) and a concomitant decrease in NAA/Cr were proposed for recurrent malignant lesions compared to necrotic areas (11–14). Beyond the use of MR spectroscopy for discrimination of radiation necrosis and recurrent tumor, several studies suggested tumor-specific metabolite profiles for distinguishing diverse brain cancers and other non-cancerous cerebral diseases (15–18). A combination of elevated Cho, decreased Cr and NAA as well as prominent lipid/lactate levels were considered to be characteristic for brain metastases (19–22). Furthermore, high lactate levels have been found in late stage brain metastases, indicating worse patient prognosis (23). Pre-clinical research represents an important part of the effort to improve treatment option by allowing mechanistic insight into cellular and molecular drivers of tumor progression and by providing platforms to test the efficacy of novel therapeutic avenues. For this purpose, experimental tumor models and adequate technological tools are required to mimic the clinical situation as closely as possible and thus improve the quality and translational significance of preclinical research (24, 25). To date, several studies reported metabolic changes after radiotherapy in glioma cells as well as post-irradiation effects in mouse and rat models (26–29). However, there is a lack of a thorough investigation of metabolic changes in response to irradiation in experimental brain metastasis using devices specifically designed for preclinical research on small animals. The goal of this study was to investigate tumor progression and therapeutic response in an experimental syngeneic breast-to-brain metastasis model using MR imaging and MR spectroscopy. We employed a small animal 7 Tesla MR scanner adapted for MR spectroscopy and the Small Animal Radiation Research Platform (SARRP) (30) that allows for image-guided application of gamma radiation with an accuracy equivalent to clinical radiotherapy. Here, we demonstrate that MR spectroscopy in combination with MR imaging represents a valuable tool for non-invasive monitoring of parameters that reflect tumor progression or regression and provide detailed insight into the histopathological and metabolic status of brain metastases to evaluate therapeutic efficacy over time in a pre-clinical model.

## Materials and Methods

### Mice

All animal studies were approved by the government committee (Regierungspräsidium Darmstadt, Germany) under the protocol number F123-1016 and were conducted in accordance with the requirements of the German Animal Welfare Act. C57BL6/J mice were purchased from Charles River Laboratories, Sulzfeld, Germany or bred within the animal facility at the Georg-Speyer-Haus, Frankfurt am Main.

### Cell Lines

The brain metastatic (BrM) variant of the murine 99LN cell line was derived from a metastatic lesion in the lymph node of the MMTV; PyMT (Murine mammary tumor virus; Polyoma middle T antigen) breast cancer model and selected *in vivo* for brain homing capacity as previously described (31). The 99LN-BrM cell line was maintained in DMEM with 10% fetal bovine serum with 1% L-glutamine and 1% penicillin / streptomycin.

### Generation of Experimental Brain Metastases

For brain metastases generation in immuno-competent mice, 6 × 10^4^ 99LN-BrM cells were inoculated into the left ventricle of 10–12-week-old female C57BL6/J mice. For tissue isolation, mice were anesthetized with 180 mg/kg ketamine and 10 mg/kg xylazine, respectively. Mice were trans-cardially perfused with PBS and 4% PFA and tissue was fixed in 4% PFA for histology.

### Tissue Preparation and Immunostaining

For immunofluorescence staining, PFA fixed brain samples were sliced into 500 μm thick sections using a Vibratome VT1200S (Leica, Nussloch, Germany). Brain slices were cleared using the X-Clarity tissue clearing system (Logos Biosystem, Inc., Anyang-si, South Korea). Tissue clearance was performed at 0.6 A for 3 h using the X-Clarity electrophoretic tissue clearing solution. After tissue clearing, unspecific protein binding was blocked with 3% BSA in PBS containing 0.1% Triton-X100 and mouse-on-mouse blocking agent. Incubation of the primary antibodies rabbit anti-mouse NeuN (abcam; 1:3,000), goat anti-mouse GFAP (abcam; 1:1,000), and mouse anti-mouse γH2AX (Millipore, 1:200) was performed for 24 h at room temperature followed by incubation of fluorescently labeled secondary antibodies (Jackson Immunoresearch, 1:500) overnight at room temperature. Hoechst was used as nuclear counterstain. Samples were embedded in X-Clarity mounting media.

For immunohistochemistry, PFA fixed samples were embedded into paraffin and 5 μm sections were prepared. Formalin-fixed paraffin embedded (FFPE) samples were processed using the Leica Bond Max (Leica, Nussloch, Germany). The automated deparaffinization/rehydration, citrate-buffer-based antigen retrieval, and blocking of unspecific protein binding and endogenous peroxidase was followed by incubation with rabbit anti-mouse EpCAM (abcam; 1:1,000), rabbit anti-Iba1 (abcam; 1:1,000), goat anti-GFAP (abcam; 1:1,000), rabbit anti-NeuN (abcam; 1:3,000), rabbit anti-Olig2 (1:500), and subsequent HRP-labeled secondary antibody incubation (Jackson Immunoresearch; 1:1,000) and incubation with the peroxidase substrate DAB. Hematoxylin was used as nuclear counterstain.

### Microscopy and Image Analysis

Immunofluorescence staining on cleared thick sections was visualized using the Yokogawa CQ1 confocal microscope (Yokogawa, Musashino, Japan) using a 60x objective.

Immunohistochemical stainings of FFPE samples were acquired by the Aperio Digital Pathology Slide scanner for gross images and a Carl Zeiss AxioImager Z1 microscope for higher magnification. Ki67-positive proliferating cells and CC3-positive apoptotic cells were quantified using the Aperio Image Scope software as percentage of total cells in the tumor area.

### Radiotherapy With the Small Animal Radiation Research Platform (SARRP)

After detection of metastatic lesions by MRI 1 day before radiotherapy (d-1) in order to determine initial values, animals were stratified into no-IR control and WBRT treatment groups. Radiotherapy was applied on d0 with the Small Animal Radiation Research Platform (SARRP, X-Strahl Ltd, Camberley, UK) (30). The SARRP is equipped with an on-board Cone Beam CT (CBCT) system for diagnostic imaging and radiation treatment process. The integrated Muriplan software allows contouring, electron density assignment, treatment planning, dose calculation, and treatment delivery. Mice were anesthetized with isoflurane (2.5%), stabilized in the prone position and imaged by performing a CBCT operating at 60 kV and 0.8 mA. CBCT images were next transferred to the Muriplan software and individual isocenters were selected for radiotherapy. Irradiation was applied as WBRT with a single dose of 10 Gy using a 10 × 10 mm collimator as 1 arc operating at 220 kV and 13 mA with 5.2 cGy sec^−1^.

### MR Imaging and Data Analysis

MR spectroscopy and MR imaging were performed using a 7 Tesla Small Animal MR Scanner (PharmaScan, Bruker, Ettlingen, Germany) with a volume coil as transmitter and a head surface coil for signal reception. Data acquisition was performed using the Paravision 6.0.1 software. For MR scans, mice were anesthetized with isoflurane (2%) and stabilized in the prone position with a tooth holder. During the MR scanning the anesthesia with ~2% of isoflurane was continued by using a built-in nosecone specially constructed for gas anesthesia. Respiration rate was monitored and kept constant at 80–100 breaths per minute continuously throughout the measurement by up- or downregulating the gas anesthesia according to the registrated respiration rate. Body temperature was maintained at 36–37°C with a built-in animal waterbed (Bruker, Ettlingen, Germany). Mice were injected intraperitoneally (i.p.) with 150 μl Gadobutrol (Gadovist, 1 mmol ml^−1^) before the measurement. Images were acquired in coronal planes. For T2-weighted images, a localized T2-multislice Turbo rapid acquisition with relaxation enhancement (T2 TurboRARE; TE/TR = 33 ms/2,500 ms) was used while a T1-weighted RARE sequence (T1 RARE; TE/TR = 6.5 ms/1,500 ms) was applied for obtaining T1-weighted images. The following other imaging parameters were used: FOV = 20 × 20 mm, 11 slices, 0.5 mm slice thickness, acquisition matrix = 256 × 256, flip angle 90°. MR images were exported as DICOM files and analyzed in OsiriX (32). Volumetric analysis of brain metastases was performed on MR image DICOM files using the threshold-based semi-automated segmentation tool (Chimaera GmbH, Erlangen, Germany) as an aycan OsiriX plug-in (33). For quantification of contrast-enhancing lesions in T1-weighted images, the high intensity smart brush tool was used given the hyperintense appearance of tumor area compared to normal brain parenchyma. Other pathologies leading to hyperintensity in MRI, such as edema could be excluded from the quantification process by comparing the MR image with its corresponding histology. After marking the tumor region with the brush, volume in mm^3^ was calculated automatically by the software plug-in.

### ^1^H MR Spectroscopy and Data Analysis

Single voxel spectroscopy (SVS) was performed using the point resolved spectroscopy sequence (PRESS) with an echo time (TE) of 16.5 ms, a repetition time (TR) of 2,500 ms, spectral width of 3301.6 Hz and 2,048 points data size. 64 acquisitions were averaged in 2:40 min. Additionally, the sequence was repeated with a TE of 135 ms, TR of 2,500 ms and 256 averages (acquisition time 10:40 min) for a lactate peak inversion. A voxel of 3 × 3 × 3 mm was placed at the brain metastases area. The target selection was based on T2-weighted MRI data from the imaging protocol.

The volume of interest (VOI) was selected to contain the brain metastases area. In order to obtain best shimming results and least disturbance by the skull as well as by tissues other than the brain, only mice with tumor lesions in the central region of the brain were used, while animals with brain metastases in the olfactory bulb or cerebellum were not included. For each measurement, homogeneity after shimming, measured as the full width at half maximum (FWHM) of the water peak was below 25 Hz. MR spectroscopic data was processed using the java-based MR user interface spectroscopic analysis package (jMRUI version 5.2) (34) employing AQSES (Automated Quantification of Short time Echo MRS), a time domain quantification method with finite impulse response (FIR) filtering, with which the residual water component could be filtered during the post-processing. Additionally, Cramer Rao lower bounds (CRLBs) of the metabolites of interest could be obtained from the quantification procedure (35). The following metabolites were included in the analysis: alanine (Ala), aspartate (Asp), ascorbate/vitamin C (Asc), glycerophosphocholine (GPC), phosphocholine (PCho), creatine (Cr), phosphocreatine (PCr), glucose (Glc), glutamine (Gln), glutamate (Glu), glutathione (GSH), glycine (Gly), myo-inositol (myo-Ins), lactate (Lac), N-acetylaspartate (NAA), N-acetylaspartylglutamate (NAAG), phosphoethanolamine (PE), scyllo-inositol (scyllo-Ins) and taurine (Tau). The respective basis data sets were generated by quantum-mechanical simulation using NMR-SCOPE-B (36), which is provided in the jMRUI package. Chemical shifts and scalar coupling constants were obtained from Govindaraju et al. (37). Based on literature, the following terms were defined to determine aggregated values for metabolites that are difficult to separate from each other: tCho = GPC+PCho, tCr = PCr+Cr, tNAA = NAA+NAAG. These values as well as Lac were considered as the most relevant for this study, and ratios were calculated for tCho/tCr, tNAA/tCr, tCho/tNAA, and Lac/tCr. MR data from non-treated tumor-free controls (*n* = 3), non-treated tumor controls (*n* = 4), irradiated tumor-free mice (*n* = 3) as well as irradiated tumor mice (*n* = 5) were obtained one day prior to irradiation and on day 2, 5, 7, 9, 12, 14, 16, 19, and 21 after irradiation. Day 21 was determined as an endpoint due to the extent of the tumor, especially in untreated tumor-bearing mice, accompanied by development of symptoms including weight loss and disequilibrium.

### Data Presentation and Statistical Analysis

Data are presented as means with standard deviation (s.d.) using GraphPad Prism Pro7. Numeric data were analyzed using two-way ANOVA and unpaired two-tailed Student's *t*-test unless otherwise noted. Differences in tumor progression were calculated as area under the curve with a non-linear curve fit for tumor growth. For comparison of the post-irradiation progression of metabolites and metabolite ratios, linear regression lines including a test for significance between the regression slopes were calculated. Baseline was corrected in all metabolites and metabolite ratios as percent deviation from the mean.

## Results

### Magnetic Resonance Imaging of Experimental Brain Metastasis

**Figure 1:**
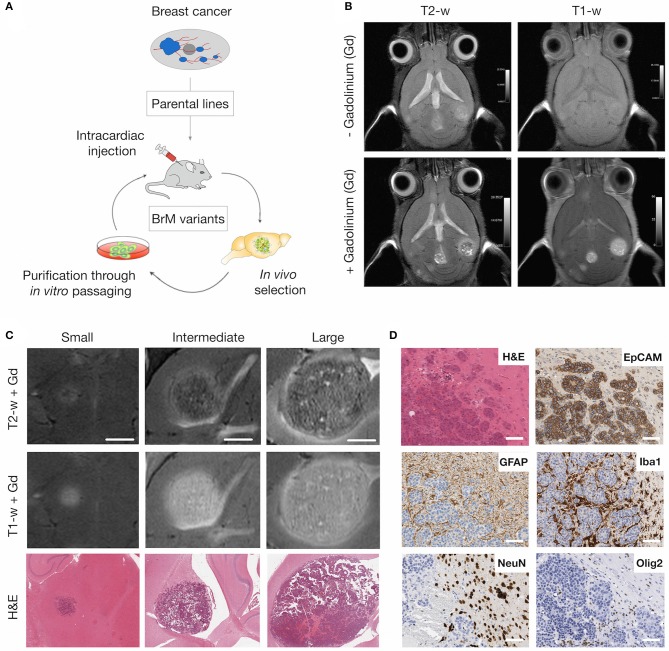
MRI of brain metastatic lesions in the 99LN-BrM model. **(A)** Schematic depicting the selection of brain metastatic variants derived from parental cell lines through multiple rounds of *in vivo* selection following intra-cardiac injection. **(B)** Representative MR images of T1- and T2-weighted sequences with and without contrast enhancement. **(C)** Representative MR images and H&E stained sections for different stages of tumor progression (small, intermediate, and large BrM). Scale bar indicates 1.5 mm. **(D)** Representative images of brain metastasis-associated stromal reactions. Tumor cells stained for the epithelial cell marker EpCAM, astrocytes, macrophages/microglia, neurons and oligodendrocytes are detected by GFAP, Iba1, NeuN, and Olig2 expression, respectively. Scale bars indicate 100 μm.

To monitor tumor progression and radio-response in experimental brain metastasis, we used an immuno-competent mouse model for breast-to-brain metastasis (Figure 1A). In this model, the brain-homing variant of the murine breast cancer cell line 99LN was injected intra-cardially (i.c.) into C57BL6/J mice, resulting in the formation of multiple metastatic lesions in the brain parenchyma (31). MR imaging using T2- and T1-weighted sequences revealed that first metastatic lesions occur after 6–8 weeks. Small lesions were detectable as hyperintense areas in T1-weighted sequences following contrast enhancement with the gadolinium-based contrast agent Gadobutrol (Figures 1B,C). In particular, T2-weighted sequences with contrast enhancement provided information on histopathological parameters. Necrosis presented as hypointense areas, while hemorrhagic areas and edema around metastatic lesions appeared hyperintense (Figure 1C). Tumor progression could be followed for 12–15 weeks after tumor cell inoculation, a time point at which most animals developed symptoms characterized by neurological deficits including head tilts and disequilibrium. Moreover, metastatic progression led to a pronounced stromal reaction characterized by the induction of astrogliosis and recruitment and activation of tumor-associated macrophages/microglia as previously described for the 99LN-BrM model and other experimental brain metastasis models (31, 38–40). In contrast to reactive astrocytes and microglia, other brain-resident cell types including neurons and oligodendrocytes were rather displaced by growing lesions (Figures 1D, **7B** for gross overview), leading to a complete absence of those cell types within metastatic lesions.

### Whole Brain Radiotherapy Induces Double Strand Breaks in Brain Resident Cell Types in Tumor-Free Mice

**Figure 2:**
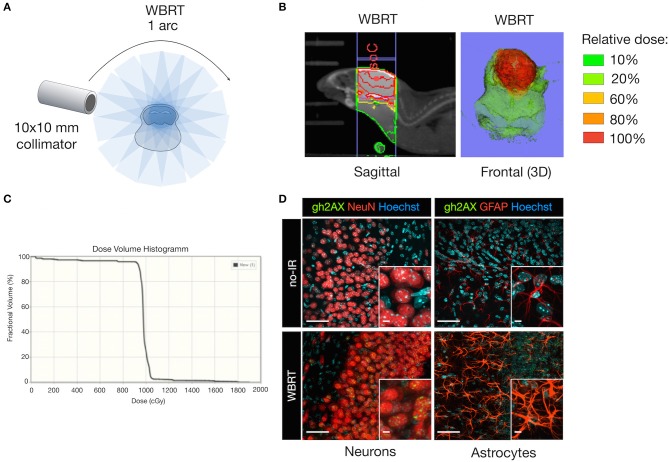
DNA damage in brain-resident cell types in response to irradiation. **(A)** Schematic depicting the treatment planning of whole brain radiotherapy (WBRT) using the 10 × 10 mm collimator. Radiation was applied as a single dose of 10 Gy using 1 arc. **(B)** Representative image of the dose distribution in the target region. The isocenter (IsoC) is depicted by the red dot. Color shades visualize the distribution of the relative dose. Shown is a sagittal view (left panel) and a three-dimensional (3D) reconstruction of the frontal view. **(C)** Dose volume histogram indicating the percentage of fractional volume over the radiation dose in cGy. **(D)** Representative images of γH2AX expression (green, lower panel) in NeuN+ neurons (red; left lower panel), GFAP+ astrocytes (red; right lower panel) on cleared thick brain sections 6 h after WBRT. Controls without WBRT are shown in the upper panel. Hoechst was used as nuclear counterstain. Scale bars indicate 50 and 5 μm.

We chose Whole Brain Radiotherapy (WBRT) as therapeutic intervention mirroring the current clinical therapy regimen for patients with multiple metastatic lesions. WBRT was applied by the SARRP (30) as a single dose (1 × 10 Gy; 1 arc) using a 10 × 10 mm collimator to cover the area of the brain but spare extracranial regions (Figure 2A). Application of WBRT using 1 arc resulted in a focused dose distribution with nearly 100% of the relative dose reaching the target organ and only marginal scattering of radiation to extracranial regions (Figures 2B,C). Next, we evaluated effects of ionizing radiation (IR) on brain-resident cell types in non-tumor bearing mice to validate that IR delivered by the SARRP leads to induction of DNA damage. Immunofluorescence staining of Ser139 phosphorylated histone H2AX (γH2AX) confirmed that the application of 1 × 10 Gy as WBRT on healthy brain leads to DNA double strand breaks in radiosensitive neurons, while no or only low γH2AX levels were detectable in radio-resistant cell types including astrocytes (Figure 2D).

### Whole Brain Radiotherapy Blocks Tumor Progression in Brain Metastasis-Bearing Mice

**Figure 3:**
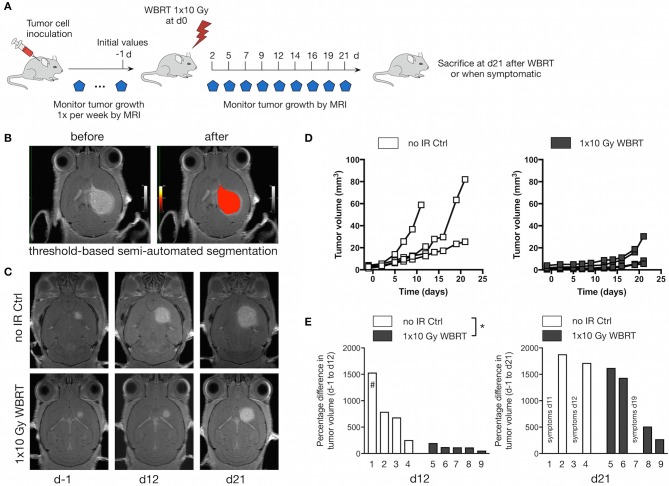
Therapeutic response to WBRT in BrM. **(A)** Schematic depicting the experimental design to follow tumor progression after WBRT in BrM. **(B)** Representative MR images showing detection and volumetric assessment of metastatic lesions using the threshold-based semi-automated segmentation tool (Chimaera GmbH). **(C)** Representative MR images of tumor-bearing mice with and without WBRT at different time points. **(D)** Quantification of the increase in tumor volume over time in tumor-bearing no-IR control mice (*n* = 4) and in response to 1 × 10 Gy WBRT (*n* = 5) using MRI. Curves depict tumor growth of individual mice. **(E)** Waterfall plots depict the percent increase in tumor volume on d12 and d21 relative to d-1. #Data shown for Mouse 1 were acquired on d11. *P*-values were obtained using two-way ANOVA and unpaired two-sided Student's *t*-test. *P < 0.05.

After we confirmed that 1 × 10 Gy WBRT leads to DNA damage in radio-sensitive brain-resident cell types (i.e., neurons), we irradiated brain metastases-bearing animals and followed the effects of WBRT over 21 days with MRI measurements every 2–3 days (Figure 3A). Volumetric analysis was performed using a threshold-based semi-automated segmentation tool (33) to detect contrast-enhancing lesions in T1 (Figure 3B). Untreated control mice (no-IR Ctrl) showed continuous tumor growth leading to an average increase in tumor volume of 816% ± 256 at d12 (*n* = 4). In contrast, WBRT led to significant reduction in the growth kinetic leading to an average increase in tumor volume of 122% ± 22 at d12 after treatment (*n* = 5) (Figures 3C–E). After the initial phase of growth stasis, tumors rebound around d16 after WBRT. To further confirm the effect of IR on the growth kinetic in the 99LN-BrM model, we compared the tumor progression and radio-response in an independent cohort with measurements once per week until d21 and observed similar effect on tumor growth as in the cohort used for spectroscopic analysis (Supplementary Figure 1A). Tumor progression in the independent cohort (*n* = 17 and 11 for untreated and treated mice, respectively) was followed until the animals developed symptoms due to brain metastasis. Survival analysis revealed that the initial effects on tumor growth do not translate into a significantly prolonged overall survival of the mice (Supplementary Figure 1B).

### Brain Metastasis Progression and Radio-Response Are Characterized by Distinct Metabolic Profiles

**Figure 4:**
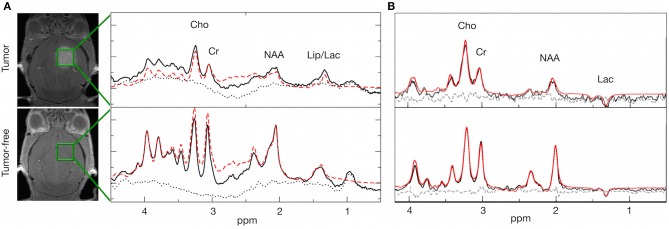
Spectral changes in response to tumor progression and radiotherapy. **(A)** Representative spectra of a tumor bearing and a tumor-free mouse with peaks for Cho (3.20 ppm), Cr (3.03 ppm), NAA (2.01 ppm), and Lac (1.33 ppm) + Lipids at short echo time (16.5 ms). **(B)** Representative spectra of a tumor bearing and a tumor-free mouse with peaks for Cho (3.20 ppm), Cr (3.03 ppm), NAA (2.01 ppm), and Lac (1.33 ppm) at long echo time (135 ms). Black line: original spectrum, red (dashed) line: spectral fit, black dotted line: residual data. Representative MR images depict the localization of the voxel.

**Figure 5:**
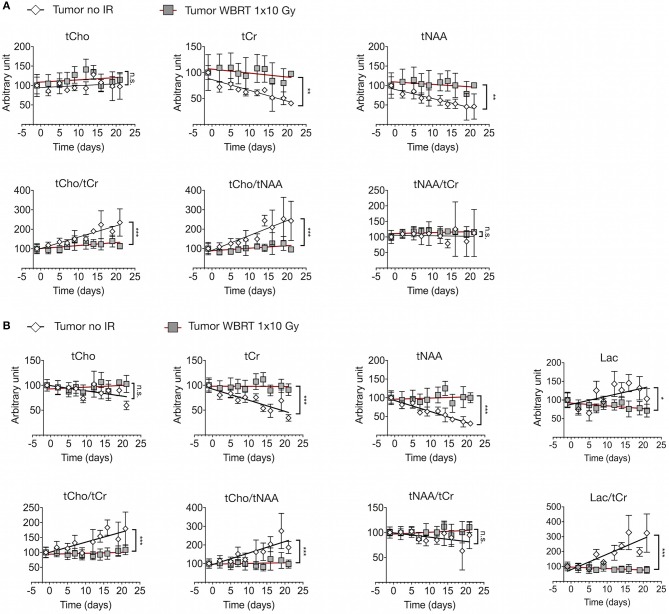
Metabolic changes in response to tumor progression and radiotherapy **(A)** Signal intensity of tCho, tCr, and tNAA (upper panel) and ratio of tCho/tCr, tCho/tNAA, and tNAA/tCr (lower panel) in tumor-bearing untreated mice (*n* = 4) and tumor-bearing mice with WBRT (*n* = 3) at short echo time (16.5 ms) (depicted as arbitrary units). **(B)** Signal intensity of tCho, tCr, tNAA and Lac (upper panel) and ratio of tCho/tCr, tCho/tNAA, tNAA/tCr, and Lac/tCr (lower panel) in tumor-bearing untreated mice (*n* = 3) and tumor-bearing mice with WBRT (*n* = 3) at long echo time (135 ms) (depicted as arbitrary units). *P*-values were calculated based on linear regression analysis and comparison between the slopes. n.s. = not significant, *P < 0.05, **P < 0.01, ***P < 0.001.

**Figure 6:**
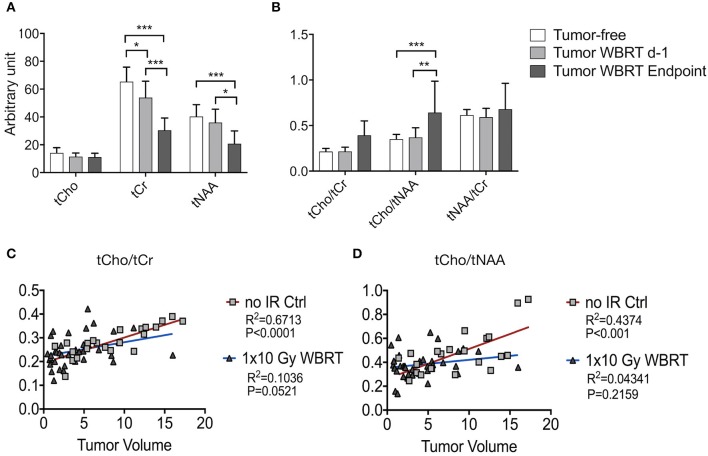
MRS data and its correlation with volumetric measurements by MRI **(A,B)** Signal intensity of tCho, tCr, tNAA **(A)** and ratio of tCho/tCr, tCho/tNAA, and tNAA/tCr **(B)** in wild-type mice and tumor-bearing mice at d-1 before WBRT and in end-stage tumors (depicted as arbitrary units). **(C,D)** Correlation between tumor volume and metabolite levels for untreated tumor bearing animals and after WBRT. Shown are graphs for tCho/tCr **(C)**, tCho/tNAA **(D)** and tumor size ranging from 0.5 to 20 mm^3^. Data are presented as mean ± s.d. *P*-values were obtained using two-way ANOVA and unpaired two-sided Student's *t*-test. Correlation between tumor volume and metabolic parameter was determined by linear regression coefficient and goodness of fit is presented as *R*^2^. n.s. = not significant, *P < 0.05, **P < 0.01, ***P < 0.001.

We next sought to assess metabolic changes during brain metastasis growth to determine metabolite concentrations at different stages of tumor progression (Figures 4, 5). We measured concentrations of creatine (tCr), choline (tCho), and N-acetylaspartate (tNAA) as indicators for energy metabolism, membrane turnover and neuronal dysfunction or displacement, respectively (15, 41). Additionally, we measured lactate (Lac) employing the lactate peak inversion at long TE for distinction between lactate signals and macromolecules (Figure 4B). Cramer Rao Lower Bounds (CRLBs) of the metabolites of interest were in mean 16.97 and 8.8% of signal intensities of each metabolite in measurements with short and long echo time, respectively, indicating a high reliability of the values obtained (Supplementary Figure 2). First, we measured metabolite concentrations in tumor-free control mice (*n* = 3) that did not receive WBRT to determine variability in metabolite levels in normal brain over time in comparison to tumor-free mice that received WBRT (*n* = 3) (Supplementary Figure 3). Our data confirmed that metabolite levels in untreated tumor-free mice remain stable over time with only minor variation of metabolite levels at different time points (Supplementary Figures 3A,B). Similar effects were observed in non-tumor bearing mice that received 1 × 10 Gy WBRT (*n* = 3) (Supplementary Figures 3A,B) indicating that irradiation does not induce major changes in cellular composition or metabolic activity in brain-resident cell types with the exception of tCho that showed a marginal increase in irradiated non-tumor bearing mice in the short TE measurements (Supplementary Figures 3A,B). In contrast, brain metastasis led to a significant decrease in tCr and tNAA concentrations, while an enhancement of lactate levels could be observed in this model (Figures 5A,B, Table 1). Despite of tumor growth, tCho concentrations remained stable over time. Calculation of ratios between different metabolites, in particular tCho/tCr, tCho/tNAA, tNAA/tCr, and Lac/tCr is used in the clinic to obtain relative metabolite concentrations based on an internal reference. tCho/tCr and tCho/tNAA additionally represent cellular density (15). Our data indicate that tumor progression is represented by the ratio of tCho/tCr and tCho/tNAA as well as by Lac/tCr in the 99LN-BrM model (Figures 5A,B, Table 1). Importantly, comparison of baseline-corrected metabolite levels as well as ratios revealed that the loss of tCr and tNAA and increase in Lac in WBRT-treated animals (*n* = 3) occurred at a slower rate than in untreated tumor-bearing mice (*n* = 4) leading to changes in metabolite levels over time close to levels found in non-tumor bearing mice during the measurement period (Figures 5A,B, Supplementary Figures 3A,B). However, metabolite levels in tumors at d-1 and WBRT end-stage tumors showed significant differences of tCr and tNAA levels compared to non-tumor-bearing brains (Figures 6A,B). Metabolic changes detected by MR spectroscopy highly correlated with volumetric measurements of tumor progression in non-treated animals (Figures 6C,D). Interestingly, in irradiated animals the correlation between volumetric and metabolic changes was less pronounced suggesting that spectroscopy detects radio-responses that are not fully captured by conventional volumetric MR imaging (Figures 6C,D). Thus, the combination of MR imaging and spectroscopy provides more comprehensive insight into histopathological and metabolic parameter for reliable analysis of therapy responses in experimental brain metastasis than conventional imaging techniques alone.

**Table 1:** Metabolite and metabolite ratios (mean with s.d.) of tumor-free, 99LN-BrM and 99LN-BrM Endpoint mice for short echo time (16.5 ms) and long echo time (135 ms) measurements.

	**Tumor-free**		**99LN-BrM**		**99LN- BrM Endpoint**	
**Short TE**	**(*n* = 10)**		**(*n* = 7)**		**(*n* = 4)**	
	**Mean**	**SD**	**Mean**	**SD**	**Mean**	**SD**
tCho	14.19	3.70	11.57	2.77	11.18	2.70
tCr	65.38	10.42	54.82	12.39	30.48	8.78
tNAA	40.30	8.56	34.22	9.04	20.79	9.15
tCho/tCr	0.21	0.04	0.22	0.05	0.39	0.16
tCho/tNAA	0.35	0.06	0.37	0.10	0.64	0.35
tNAA/tCr	0.61	0.06	0.62	0.05	0.68	0.28
**Long TE**	**(*n* = 6)**		**(*n* = 6)**		**(*n* = 3)**	
	**Mean**	**SD**	**Mean**	**SD**	**Mean**	**SD**
tCho	3.62	0.42	4.23	0.40	2.61	0.35
tCr	17.75	1.73	17.37	2.88	7.97	3.16
tNAA	8.01	1.77	7.01	1.67	3.14	1.29
tCho/tCr	0.21	0.03	0.25	0.05	0.36	0.15
tCho/tNAA	0.47	0.12	0.64	0.19	0.90	0.24
tNAA/tCr	0.45	0.07	0.40	0.04	0.40	0.12
Lac	2.79	0.45	2.86	0.63	2.43	0.68
Lac/tCr	0.16	0.03	0.17	0.06	0.36	0.23

*Metabolite values are depicted in arbitrary units*.

### Assessment of Histopathological Parameters After Whole Brain Radiotherapy

**Figure 7:**
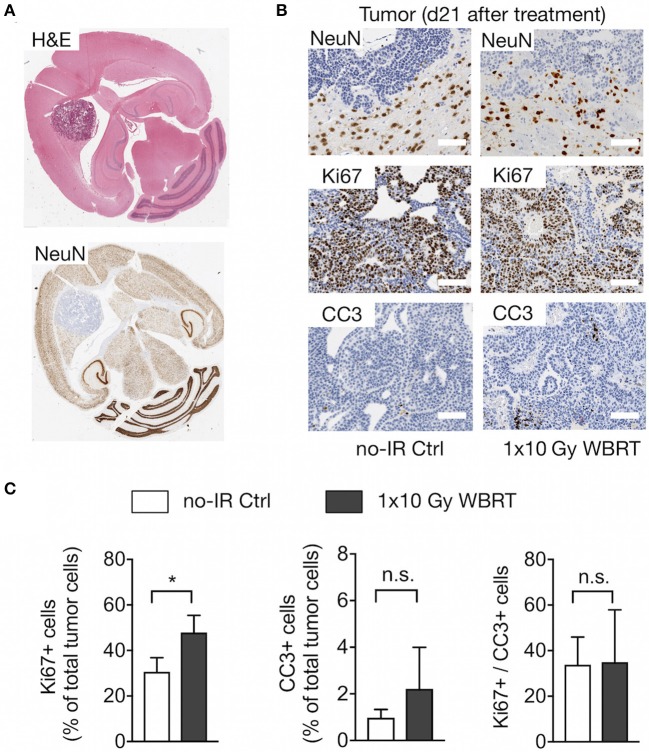
Histopathological assessment. **(A)** Overview of the localization of neurons in brain metastasis shown by H&E and NeuN staining to detect neurons. **(B)** Higher magnification images of NeuN+ neurons at the tumor-stroma interface, Ki67+ proliferating tumor cells and CC3+ apoptotic cells in brain metastatic lesions in tumor-bearing mice with and without WBRT. Scale bars indicate 100 μm. **(C)** Quantification of proliferation and apoptosis rate and the proliferation/apoptosis index in mice with and without WBRT (*n* = 4 for each group in both graphs). Data are presented as mean ± s.d.. *P*-values were obtained using unpaired two-sided Student's *t*-test. n.s. = not significant, *P < 0.05.

To validate changes in histopathological parameters detected by MR imaging and spectroscopy, we performed histological assessment of the rate of proliferation and apoptosis as well as the presence of neurons within the analyzed tumor area at the trial endpoint. Histological detection of neurons confirmed that brain metastatic progression leads to displacement of NeuN+ cells indicating that the decrease in tNAA levels can be attributed to the reduction in the number of neurons within the tumor lesions while the tumor area within the voxel increased (Figures 7A,B). Displacement of normal brain tissue occurred at a slower rate in response to WBRT leading to higher levels of tCr and tNAA. Analysis of the proliferation rate revealed a significantly higher percentage of Ki67+ cells in rebound tumors after IR compared to untreated animals at the trial endpoint with a trend toward a higher percentage of CC3+ apoptotic cells in irradiated tumors. Consequently, the ratio of Ki67+ to CC3+ cells was similar in both groups in end-stage tumors (Figures 7B,C). Moreover, histological assessment revealed the presence of necrotic areas in irradiated and control tumors at trial end point indicating that tumor progression itself leads to necrosis. Comparison of tumors with similar sizes in both groups showed a more dense growth pattern in the control tumors indicating that radiation enhances the induction of necrosis (Supplementary Figure 4) but is not the only cause for necrosis at trial endpoint.

In sum, we demonstrated that MR imaging in combination with MR spectroscopy represents a valuable tool for non-invasive, longitudinal measurement of experimental brain metastasis. Assessment of histopathological and metabolic parameter that are characteristic for progressing vs. regressing tumors are important measures to evaluate therapeutic efficacy over time.

## Discussion

Recent technological and conceptual advances provide unprecedented opportunities to identify and study drivers of brain metastasis with the ultimate goal to develop novel treatment options to improve prognosis and quality of life for brain metastases patients. The development of novel therapeutic avenues (5, 42, 43) requires thorough preclinical testing of intervention strategies in experimental models that closely mimic the clinical situation. Closing the technological gap between clinical and preclinical instrumentation and employing diagnostic tools used for patients will improve the translational significance of preclinical research (24, 30, 44). To test the applicability of implementing MR spectroscopy into the monitoring routine in the pre-clinical setting, we chose radiotherapy as the therapeutic intervention strategy. Despite the debate on WBRT concerning its long-term side effects and failure to prolong overall survival, it still remains part of current clinical therapy regimens for brain metastasis patients with short life expectancy. Data obtained by MR spectroscopy revealed a pronounced reduction of total NAA and total creatine as well as a significant increase in lactate levels in untreated 99LN-BrM-bearing mice, while total choline levels remained stable over time. We observed a strong correlation between tumor growth and reduction of tCr and tNAA as well as increase in Lac. Reduction of tNAA levels during tumor growth is indicative for the loss of neuronal structures or function (41) and gradual loss of tCr and tNAA in progressing brain tumors can at least in part be attributed to the displacement of normal brain structures by highly proliferating tumor cells. Hence, the partial volume effect is expected to be more pronounced in brain metastases with expanding growth pattern compared to brain tumors with infiltrative growth such as high-grade glioblastoma (16–18). Indeed, while NAA levels are known to be decreased in primary brain tumors such as glioblastoma and astrocytoma, it has been shown to be almost completely absent in brain metastases (45–48). In line with these findings, we found that brain metastases progression in the 99LN-BrM model leads to displacement of neurons by the growing tumor with no NeuN+ cells being detectable within the tumor bulk. At early stages of tumor progression, the volume of interest contained small metastatic lesions and a considerable amount of adjacent healthy brain tissue. The ratio of tumor area to healthy brain tissue within the voxel changed over time with less normal parenchyma contributing to the spectrum at advanced disease stages.

Creatine is considered as a marker for cellular aerobic energy metabolism given its role in ATP synthesis mediated by the creatine kinase (49). Creatine levels have been shown to be stable under different conditions (50, 51), while displaying differences in distinct brain regions (52). It was demonstrated that creatine levels are significantly lower in non-neuroectodermal tumors, i.e., in brain metastases compared to neuroectodermal tumors such as glioblastoma (11, 48, 53, 54). For example, Ishimaru et al. described a complete absence of creatine in brain metastases (16), which supports our interpretation of a partial volume effect also in creatine levels due to the expanding growth of brain metastases. Accordingly, tNAA/tCr, which is often calculated under the premise of stable creatine, is not significantly reduced over time in experimental brain metastasis. Lactate, resonating at 1.33 ppm, indicates enhanced anaerobic metabolism and is associated with brain tumor, cerebral ischemia, stroke and mitochondrial diseases, such as Mitochondrial encephalopathy, lactic acidosis, and stroke-like episodes (MELAS) or Myoclonic Epilepsy with Ragged Red Fibers (MERRF) (23, 55–58). Several studies suggested increased lactate concentrations in higher grades of brain cancer as well as in rapidly growing tumors (23, 57, 59, 60). We observed increasing levels of lactate during tumor progression in untreated mice, while it remained stable in irradiated tumor-bearing and wild-type mice. This indicates that suppression of tumor growth in response to irradiation in 99LN-BrM mice results in lower cell density and thus might lead to less production of lactic acid over time. Lactate has also been described to be enhanced in regions with subacute radiation necrosis in addition to reduced NAA levels (26, 61, 62). However, a detection of radiation necrosis in terms of increased lactate levels or reduced NAA after WBRT could not be confirmed in irradiated wild-type controls in this study. Supposably, the observation time after the irradiation was too short to detect any long-term side effects of the irradiation. It is also possible that the irradiation dose was not sufficient to cause pronounced radiation necrosis as also evidenced by histopathological assessment.

Surprisingly, we did not observe elevated choline levels in brain metastases compared to normal brain tissue and during tumor progression. This finding is inconsistent to previous findings that described significant increase of choline in all analyzed brain tumors (61, 63–65) and other solid cancer types such as breast cancer (66–68) compared to adjacent healthy tissue. Total choline compounds, consisting of phosphocholine, glycerophosphocholine, and free choline, are considered as a surrogate marker for membrane integrity and turnover (41). Moreover, an increase in total choline and a switch from glycerophosphocholine to phosphocholine has been described in malignant lesions (69–71). However, high choline concentration was also described in normal glial cells (72). It is possible that in the 99LN-BrM model, choline levels in tumor cells and glial cells are similar. Consequently, a partial volume effect by displacing normal tissue would not be detectable in changes in choline levels. Stable choline levels in tumor-bearing mice over time might also be explained by the involvement of necrotic and cystic areas with decreased choline levels (63, 65). Given the instrument configuration and the small size of brain metastases in mouse models, it is technically challenging to target only viable tumor areas in the MR spectroscopy measurements due to tumor heterogeneity. Hence, a possible increase of choline levels in viable tumor areas with high proliferation rates might be masked by the presence of dying cells in necrotic areas. Measurements in more homogeneous tumor areas would facilitate the interpretation of acquired spectra. However, selecting a smaller voxel size significantly increases measurement time and requires additional techniques such MR spectroscopic imaging (73).

Given the differences in the correlation coefficient in the no-IR Ctrl and WBRT treatment groups with respect to volumetric and metabolic measurements, our data on experimental brain metastasis indicate that MR spectroscopy detects therapeutic responses that are not fully captured by MR imaging. In future studies, we plan to include MR spectroscopy in our preclinical trials as an additional non-invasive diagnostic tool to gain insight into therapeutic responses over time to evaluate treatment efficacy of different novel intervention strategies that hopefully provide strong scientific rationale for improved therapeutic avenues for brain metastasis patients.

## Data Availability Statement

All datasets generated for this study are included in the article/Supplementary Material.

## Ethics Statement

All animal studies were approved by the government committee (Regierungspräsidium Darmstadt, Germany) under the protocol number F123-1016 and were conducted in accordance with the requirements of the German Animal Welfare Act. C57BL6/J mice were purchased from Charles River Laboratories, Sulzfeld, Germany or bred within the animal facility at the Georg-Speyer-Haus, Frankfurt am Main.

## Author Contributions

WC and LS designed the project. WC, KN, MS, JB, FR, UP, and LS performed experiments and analyzed data. MP, FK, and JJ provided material. FR, BB, and UP advised on radiotherapy and MR spectroscopy. WC and LS wrote the manuscript. All authors read and approved the manuscript.

### Conflict of Interest

The authors declare that the research was conducted in the absence of any commercial or financial relationships that could be construed as a potential conflict of interest.

## Supplementary Material

Supplementary Figure 1 Growth kinetic of 99LN-BrM tumors and overall survival in response to IR **(A)** Quantification of the tumor volume based on T1 weighted MRI images during tumor progression in the 99LN-BrM model in untreated mice (*n* = 17) and mice after WBRT (*n* = 11). **(B)** Kaplan-Meier curves show the percentage of the overall survival of 99LN-BrM-bearing untreated mice (*n* = 17) and after WBRT (*n* = 11). P-values were calculated based on area under the curve and unpaired two-sided Student's *t*-test for **(A)** and survival curves were compared using Mantel-Cox log-rank test. ^*^*P* < 0.05 and n.s., not significant.

Supplementary Figure 2 Cramer Rao Lower Bounds (CRLBs) of metabolites for short (16.5 ms; **A**) and long echo time (135 ms; **B**) measurements. Data are presented as mean ± s.d. in % of respective signal intensities.

Supplementary Figure 3 Metabolic changes in untreated tumor free control mice and tumor-free mice that received WBRT over time. **(A)** Signal intensity of tCho, tCr, and tNAA(upper panel) and ratio of tCho/tCr, tCho/tNAA, and tNAA/tCr (lower panel) in tumor free control mice (*n* = 3) and tumor-free mice that received WBRT (*n* = 3) at short echo time (16.5 ms) over time (depicted as arbitrary units). **(B)** Signal intensity of tCho, tCr, tNAA, and Lac (upper panel) and ratio of tCho/tCr, tCho/tNAA, tNAA/tCr, and Lac/tCr (lower panel) in tumor-free control mice (*n* = 3) and tumor-free mice that received WBRT (*n* = 3) at long echo time (135 ms) over time (depicted as arbitrary units). *P*-values were calculated based on linear regression analysis and comparison between the slopes. n.s., not significant, ^*^*P* < 0.05 and ^**^*P* < 0.01.

Supplementary Figure 4 Histology of 99LN-BrM tumors at trial end point. Hematoxylin and eosine (H&E) stained brain sections depict the histopathology of 99LN-BrM tumors at trial end point as gross overview and higher magnification using a 10x objective. Scale bars indicate 100 μm.
