# Supplementary figures and images for: Evaluating Magnetic Resonance Spectroscopy as a Tool for Monitoring Therapeutic Response of Whole Brain Radiotherapy in a Mouse Model for Breast-to-Brain Metastasis
Source: Front Oncol. 2019 Nov 27;9:1324. doi: 10.3389/fonc.2019.01324 (PMC6890861; doi:10.3389/fonc.2019.01324)

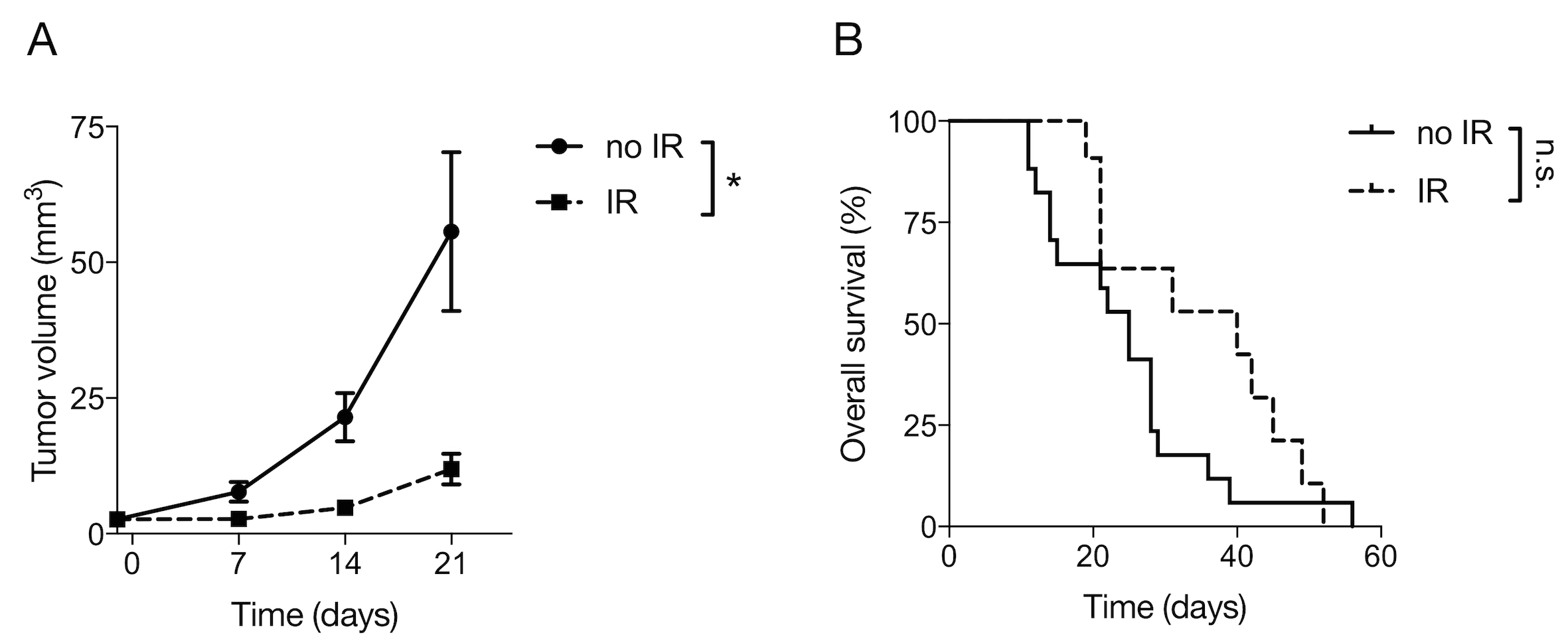

Supplement: Supplementary Figure 1 — Growth kinetic of 99LN-BrM tumors and overall survival in response to IR (A) Quantification of the tumor volume based on T1 weighted MRI images during tumor progression in the 99LN-BrM model in untreated mice (n = 17) and mice after WBRT (n = 11). (B) Kaplan-Meier curves show the percentage of the overall survival of 99LN-BrM-bearing untreated mice (n = 17) and after WBRT (n = 11). P-values were calculated based on area under the curve and unpaired two-sided Student's t-test for (A) and survival curves were compared using Mantel-Cox log-rank test. *P < 0.05 and n.s., not significant. [file Image_1.JPEG]

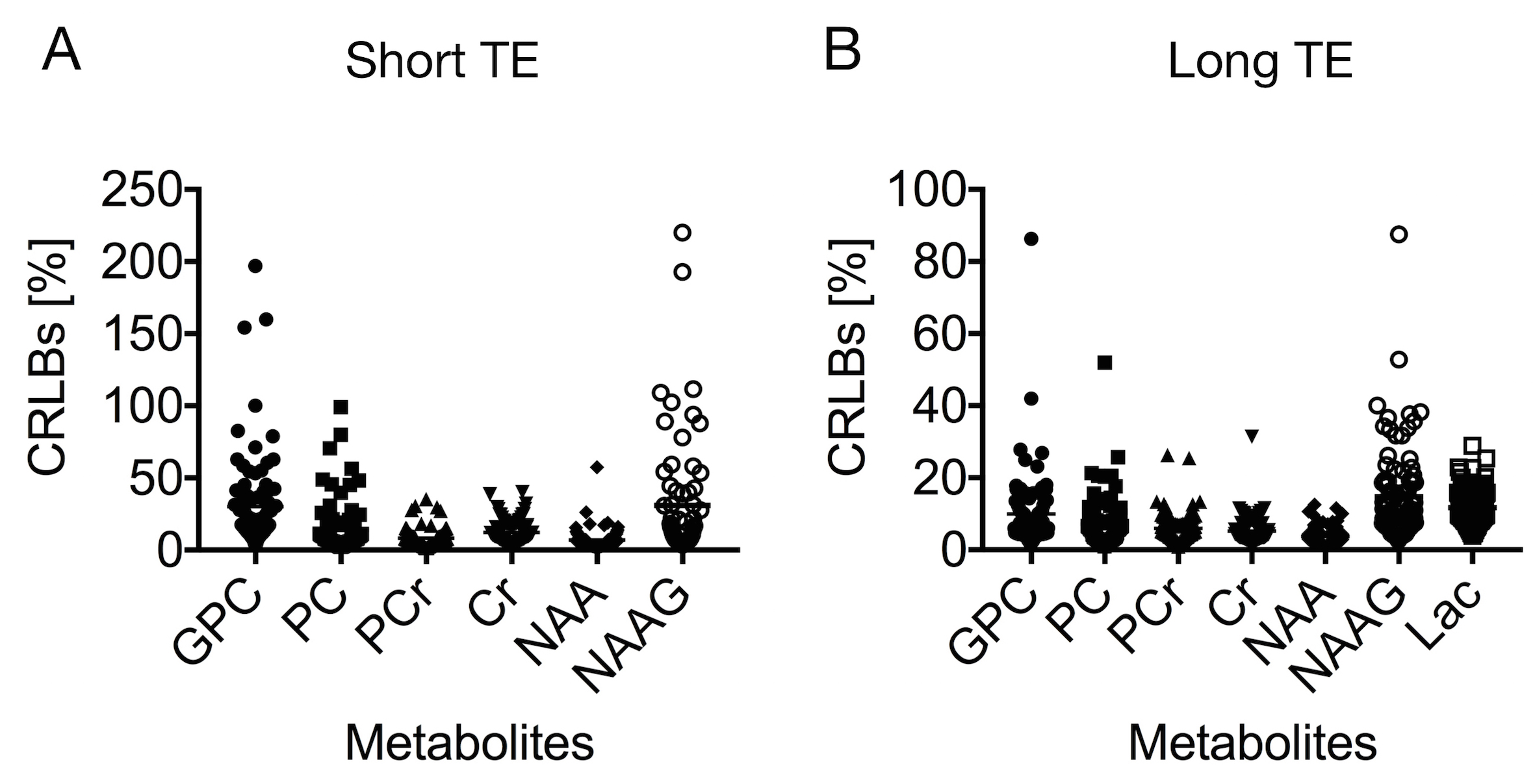

Supplement: Supplementary Figure 2 — Cramer Rao Lower Bounds (CRLBs) of metabolites for short (16.5 ms; A) and long echo time (135 ms; B) measurements. Data are presented as mean ± s.d. in % of respective signal intensities. [file Image_2.JPEG]

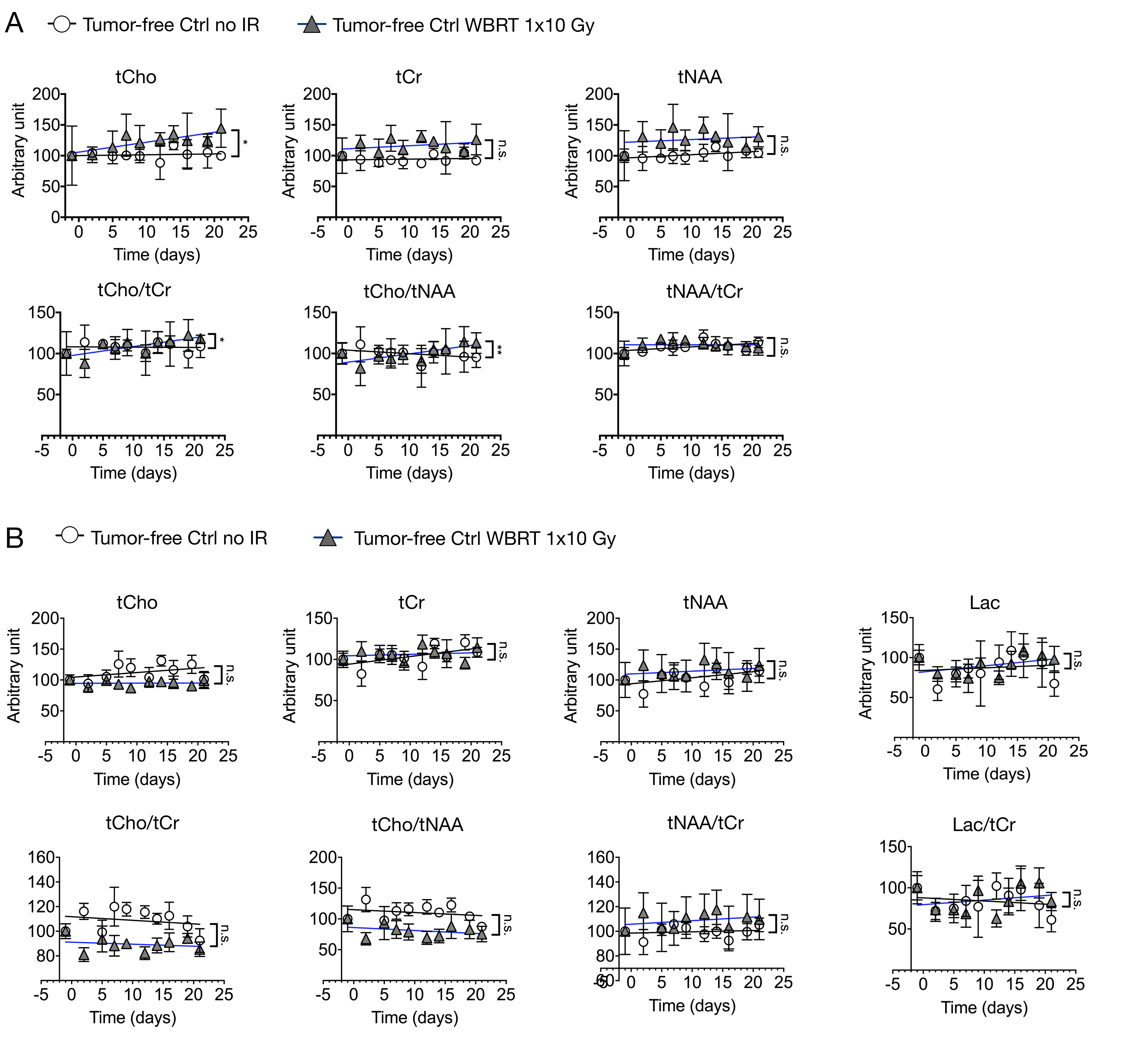

Supplement: Supplementary Figure 3 — Metabolic changes in untreated tumor free control mice and tumor-free mice that received WBRT over time. (A) Signal intensity of tCho, tCr, and tNAA(upper panel) and ratio of tCho/tCr, tCho/tNAA, and tNAA/tCr (lower panel) in tumor free control mice (n = 3) and tumor-free mice that received WBRT (n = 3) at short echo time (16.5 ms) over time (depicted as arbitrary units). (B) Signal intensity of tCho, tCr, tNAA, and Lac (upper panel) and ratio of tCho/tCr, tCho/tNAA, tNAA/tCr, and Lac/tCr (lower panel) in tumor-free control mice (n = 3) and tumor-free mice that received WBRT (n = 3) at long echo time (135 ms) over time (depicted as arbitrary units). P-values were calculated based on linear regression analysis and comparison between the slopes. n.s., not significant, *P < 0.05 and **P < 0.01. [file Image_3.JPEG]

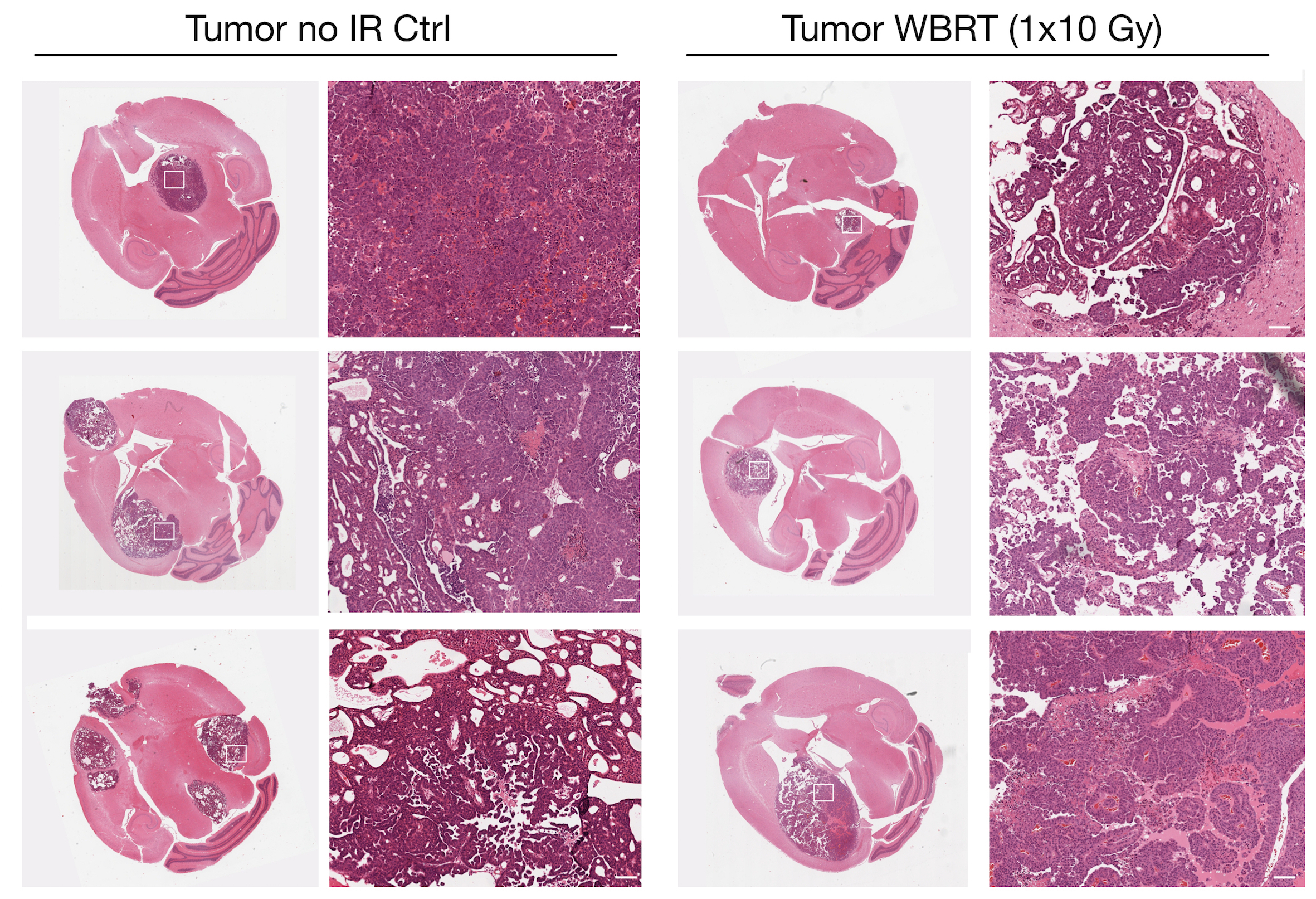

Supplement: Supplementary Figure 4 — Histology of 99LN-BrM tumors at trial end point. Hematoxylin and eosine (H&E) stained brain sections depict the histopathology of 99LN-BrM tumors at trial end point as gross overview and higher magnification using a 10x objective. Scale bars indicate 100 μm. [file Image_4.JPEG]
